# Challenges in Assessment of Health Systems Decentralization: The Role of Path Dependence and Choice of Indicators: Comment on "The Effects of Health Sector Fiscal Decentralisation on Availability, Accessibility, and Utilisation of Healthcare Services: A Panel Data Analysis"

**DOI:** 10.34172/ijhpm.2023.74274

**Published:** 2023-06-18

**Authors:** Flavia Mori Sarti

**Affiliations:** School of Arts, Sciences and Humanities, University of Sao Paulo, Sao Paulo, Brazil

**Keywords:** Health System, Decentralization, Path Dependence, Outcome Indicator, Public Health, Healthcare

## Abstract

Optimal resource allocation within national health systems represents the ultimate challenge in diverse countries worldwide. Major part of the literature points that health systems decentralization potentially address the challenge. The present commentary focuses on the debate referring to effects of health systems decentralization, based on the evidence of the study of Arianna Rotulo and colleagues. Studies on the subject emphasize the role of path dependence and the influence of choice of indicators for measurement of effects in the assessment of health systems decentralization. Acknowledging the complexity of the phenomena, the results of the study of Rotulo et al on health system decentralization in Italy are highlighted through the analysis of recent evidence from the literature. The present commentary shows that there are diverse indicators adopted in the literature on the subject, pointing to mixed results, depending on country characteristics and selection of indicators in the analysis. The synthesis of indicators gathered in recent studies also indicate that health system indicators are sensitive to path dependence, thus, requiring additional attention to assumptions of studies on health systems decentralization. Thus, studies should consider the influence of path dependence on organizational practices and institutional structures involved in decentralization processes, in addition to acknowledging that assessments on decentralization vary substantially according to indicators adopted in the analysis, and their links with previous decisions within health systems.

 The present commentary focuses on the debate regarding effects of health systems decentralization, based on evidence from the study of Arianna Rotulo and colleagues.^1^ The potential role of path dependence (ie, dependence on the history and the order of previous events^2^) and the choice of indicators for measurement of decentralization effects are emphasized in the commentary to identify diverse approaches in the assessment of health systems decentralization.

 Optimal resource allocation within national health systems represents the ultimate challenge in diverse countries worldwide. Major part of the literature points that health systems decentralization potentially address the challenge, particularly in countries with substantial intervention of the public sector, considering that the provision of merit goods may benefit from supply at local level due to in-depth knowledge of population needs and preferences.^3,4^

 Yet, evidence from studies assessing health systems decentralization during recent decades identifies mixed results, depending on country characteristics and selection of indicators. Rotulo et al^1^ showed that fiscal decentralization may negatively impact health services due to intensification of existing inequalities in the distribution of resources within the country, through decrease of healthcare availability, accessibility, and utilisation. According to the authors,^1^ the allocation of pooling and expenditures to local governments aggravated disparities among Italian regions from 2001 until 2017, showing decrease in access and availability of resources in public hospitals, whilst increasing resources in private healthcare settings.

 Considering elements in the literature of health systems decentralization, authors should ponder the influence of path dependence and choice of indicators for assessment of decentralization effects.^1^ Acknowledging the complexity of decentralization phenomena, Rotulo et al^1^ highlight negative effects of fiscal decentralization of the Italian health system through the analysis of selected indicators in the context of Italy, a high-income country.

 First, concerning path dependence, authors emphasize historical inequalities among country regions, and underscore that the design of the Italian National Health Service (*Servizio Sanitario Nazionale*) was intended towards promotion of universal healthcare access (ie, assurance of healthcare for the whole population independently of individuals’ personal and health characteristics), funded through federal taxes and operationalized at local level. Fiscal decentralization occurred in 2001, including services restructuration and additional financing at local level with co-payment.^1^

 Second, regarding choice of indicators, the results obtained should be interpreted with caution due to certain limitations in variables included in the analysis. Rotulo et al^1^ identify indicators in three dimensions of the Italian health system: healthcare availability, accessibility, and utilization.

 Healthcare availability was represented by indicators of human resources and diagnosis equipment in public and private settings.^1^ However, the latter comprise indicator with dubious interpretation due to its intrinsic characteristics: being diagnosis equipment with high fixed costs, potentially reduces the occurrence of severe health problems, and is probably used for diagnosis of patients residing in diverse regions.

 Healthcare accessibility was represented by proportion of hospitalizations of individuals residing in the hospital vicinity, potentially including diverse medium to high complexity treatments. Considering high fixed costs of medium to high complexity healthcare, there is usually concentration of facilities in certain regions, attracting patients from regions without specialized healthcare, which biases the indicator. In addition, strategic actions of preventive care may also influence the indicator: regions focusing on preventive measures potentially reduce healthcare needs of their inhabitants regarding medium to high complexity procedures.

 Healthcare utilization was represented by hospitalizations in public and private hospitals, potentially including premature discharges, which may lead to negative health outcomes, including complications, unplanned readmissions, and higher mortality.^5^ In addition, the indicator may be influenced by adoption of preventive care (reducing hospitalizations) or increase in unplanned readmissions (increasing early mortality).

 Therefore, part of the indicators in the study of Rotulo et al^1^ should consider interactions with other variables, since it is important to acknowledge that prevention represents lower costs and higher quality of life for individuals and populations, whilst curative tertiary level interventions result in higher costs and potential detrimental impacts on individuals’ health and wellbeing.

 Finally, the potential effects of decentralization referring to expansion of private sector in low-income regions^1^ may be linked to either lower costs or higher quality of private healthcare (in comparison to public sector), or to higher public sector expenditures in prevention strategies in high-income regions, leading to improvements in population health outcomes.

 Contradicting the results from Rotulo et al,^1^ Costa-Font and Turati^6^ showed that inequalities in outcomes and outputs in Italian and Spanish health systems were associated to differences in design and management of health services by local governments, instead of healthcare decentralization processes. However, the authors adopted different measures of regional inequality output and health system outcome with different empirical strategy, based on concentration indexes, for the analysis of pre- and post-decentralization inequalities within national health systems.

 De Siano and D’Uva^7^ indicated potential opportunistic behaviour of local governments to benefit from spillovers from public expenditures of neighbour regions in the process of decentralization in Italy from 1996 to 2010. In addition, authors point to economies of scale, shift of demand to the private sector, and movement of individuals to regions with higher income due to unsatisfaction with local health services. The authors focused on levels of public expenditures in three sectors (health, education, and transport) using spatial Durbin model to assess the impact of decentralization on spillover effects.^7^

 Major part of studies on health systems decentralization focuses on indicators of responsiveness, efficiency, and effectiveness in healthcare delivery.^8^ Evidence from studies on health system decentralization in other countries reinforce divergences according to country characteristics and type of indicator. Health system decentralization has been usually associated with beneficial results in primary healthcare in developed and developing countries,^9,10^ which are related to health promotion and disease prevention at lower costs, and associated with long term beneficial health outcomes for the population.

 Cobos Muñoz et al^11^ showed that decentralization of governance, financing, and healthcare delivery generally present positive effects, whilst resource management decentralization may have negative effects. According to evidence from 30 European countries from 1996 to 2015, the effects of decentralization of healthcare expenditures were negative on populations’ perceptions of healthcare quality.^12^

 Dwicaksono and Fox^13^ performed a systematic review of literature, showing mixed results regarding the effects of decentralization on health system indicators, although effects on indicators of system performance and health outcomes were generally positive. However, it is important to highlight that the authors assessed the quality of studies included in the systematic review, considering only 10 studies with low risk of bias.

 In addition, the decentralization process may present different effects in developing countries compared to developed countries, emphasizing the role of path dependence in the evolution of organizational practices and institutional arrangements within diverse structures of incentives. Abimbola et al^14^ synthesized quantitative and qualitative evidence on the effects of health systems decentralization in low-, medium- and high-income countries, indicating mechanisms and contextual factors at institutional, socioeconomic, and geographic levels influencing decentralization results on equity, efficiency, and resilience in healthcare.

 The authors emphasize the complexity of the decentralization phenomena, showing that national and local government characteristics may influence the results of health systems decentralization. Therefore, decentralization may generate competition, cooperation, or coopetition (competition and cooperation) among organizations and governments through creation of multiple governance centres with vertical and horizontal relations amongst themselves.^14^

 In relation to the study of Rotulo et al,^1^ the decentralization process in the Italian health system follows the path of aggravating previous patterns of inequities through the mechanism of ‘voting with feet,’ phenomena based on the migration of individuals and resources from low- to high-income locations.^13^

 Thus, previous inequities in distribution of resources among local governments play important role in determining the capacity of provision of public services, contributing to divergences in results of health system decentralization.^9^ Recent evidence indicates that the process of decentralization may present important trade-offs between economies of scale, transaction costs and agency costs due to bureaucracy. In addition, lack of public sector accountability may support the capture of political power by local elites, jeopardizing efficiency, efficacy, and outcomes in local health systems.

 On the one hand, decentralization may generate disproportionate bureaucracy whilst pursuing improvements in quality, safety, and efficiency of healthcare delivery; on the other hand, health system centralization may lead to imposition of ineffective patterns for provision of health services at national level, depending on country characteristics.^15^

 In fact, Costa-Font and Greer^16^ argue that health systems decentralization comprises diverse processes referring to various models of delegation or distribution of activities regarding organization, financing, management, and delivery of healthcare within different socioeconomic, cultural, and political backgrounds. Consequently, assorted degrees of responsiveness, efficiency, and effectiveness should be expected accordingly, especially considering the fundamental role of organizational practices and institutional structures in determining incentives and accountability to achieve optimal performance whilst minimizing risks of principal-agent problems.^17^

 The argument of Costa-Font and Greer^16^ is supported by the synthesis of evidence on indicators gathered from studies previously discussed, reinforcing the requirement for careful consideration on the assumptions adopted in studies assessing health systems decentralization (Table).

**Table:** Health Systems Dimensions, Indicators, and Assumptions in Selected Studies on Health System Decentralization

**Health System Dimensions**	**Indicators**	**Assumptions on Indicator Representativeness**
Accessibility	Hospitalised patients residing in hospital vicinity^1^	Acceptability of local healthcare supply by residents^1^
	Closeness to patients^11^	Indicator related to one of the “building blocks” of health systems defined by the WHO (service delivery)^11^
Availability	Human resources and equipment^1^	Measure of potential to provide health assistance for the population^1^
	Health services coverage^13^	Performance of healthcare due to decisions on allocation of health system inputs^13^
Efficiency	Input-output or input-outcome ratios in the health system^14^	One of the three health system goals potentially subject to trade-offs in health system decentralization processes^14^
Equity	Minimization of unnecessary and avoidable disparities in health outcomes^14^	One of the three health system goals potentially subject to trade-offs in health system decentralization processes^14^
Governance	Community participation, adaptation of planning processes to local settings, among others^11^	One of the “building blocks” of health systems defined by the WHO^11^
Inputs	Health expenditures, equipment, physical resources, and management and retention of human resources^11^	Resources necessary for healthcare delivery, representing three of the “building blocks” of health systems defined by the WHO (financing, consumables, and health workforce)^11^
	Financial, human, and physical resources^13^	Healthcare inputs defined by local governments’ choice of actions^13^
Outcomes	Satisfaction with healthcare^6^	Measurable dimension of outcome from healthcare activity^6^
	Infant mortality rates^9^	Proxy of population health indicator sensitive to policy reforms
	Type 2 diabetes mellitus mortality and morbidity^10^	Effects of autonomy in local governments decisions on population health^10^
	Mortality, relative risk of death, and post-neonatal mortality^11^	Health indicators sensitive to changes in one of the “building blocks” of health systems defined by the WHO^11^
	Perception on the quality of public services^12^	Improvements of social welfare^12^
	Health services quality^13^	Performance of healthcare due to decisions on health system inputs^13^
	Mortality rates and life expectancy^13^	Individual or population-level effects of health system performance^13^
Health system dimensions	Indicators	Assumptions on indicator representativeness of changes due to health system decentralization
Outputs	Health expenditure per capita^6^	Monetary input required for healthcare activity^6^
	Public expenditure in health^7^	Spillover effect due to possibility of citizens to benefit from public services supplied by neighbour governments^7^
	Performance of healthcare, quality of health information, among others^11^	Outputs related to two of the “building blocks” of health systems defined by the WHO (health information, and service delivery)^11^
Resilience	Adaptability and robustness of the health system to changes^14^	One of the three health system goals potentially subject to trade-offs in health system decentralization processes^14^
Utilization	Discharge rates in public and private hospitals^1^	Local governments’ policy to minimize hospital admissions (ie, local healthcare expenditures)^1^
	Healthcare utilization, among others^11^	Indicator related to one of the “building blocks” of health systems defined by the WHO (service delivery)^11^
	Health services utilization^13^	Performance of healthcare due to decisions on health system inputs^13^

Abbreviation: WHO, World Health Organization.

 Ultimately, studies should consider that assessments on health systems decentralization vary substantially according to indicators adopted in the analysis, also acknowledging their own sensitivity to path dependence, in addition to its influence on organizational practices and institutional structures involved in decentralization processes.

## Ethical issues

 Not applicable.

## Competing interests

 Author declares that she has no competing interests.

## Funding

 The present study was supported by the Brazilian Ministry of Science and Technology (Conselho Nacional de Desenvolvimento Científico e Tecnológico, productivity scholarship process 301109/2019-2).
